# Crack kinking at the tip of a mode I crack in an orthotropic solid

**DOI:** 10.1007/s10704-017-0227-x

**Published:** 2017-07-11

**Authors:** Harika C. Tankasala, Vikram S. Deshpande, Norman A. Fleck

**Affiliations:** Department of Engineering, Cambridge University, Trumpington St., Cambridge, CB2 1PZ UK

**Keywords:** Crack kinking, Orthotropy, Cohesive zone, Brittle fracture

## Abstract

The competition between crack penetration and crack kinking is addressed for a mode I macroscopic crack in an orthotropic elastic solid. Cohesive zones of finite peak strength and finite toughness are placed directly ahead of and orthogonal to the plane of the parent crack. The cohesive zone ahead of the crack tip is tensile in nature and leads to crack penetration, whereas the inclined zones slide without opening under a combined shear and normal traction, and give crack kinking. Thereby, the competition between continued crack growth by penetration ahead of the crack tip versus kinking is determined as a function of the relative strength and relative toughness of the cohesive zones. This competition is plotted in the form of a failure mechanism map, with the role of material orthotropy emphasized. Synergistic toughening is observed, whereby the parent crack tip is shielded by the activation of both the tensile and shear (kinking) cohesive zones, and the macroscopic toughness is elevated. The study is used to assess the degree to which various classes of composite have the tendency to undergo kinking.

## Introduction

**Fig. 1:**
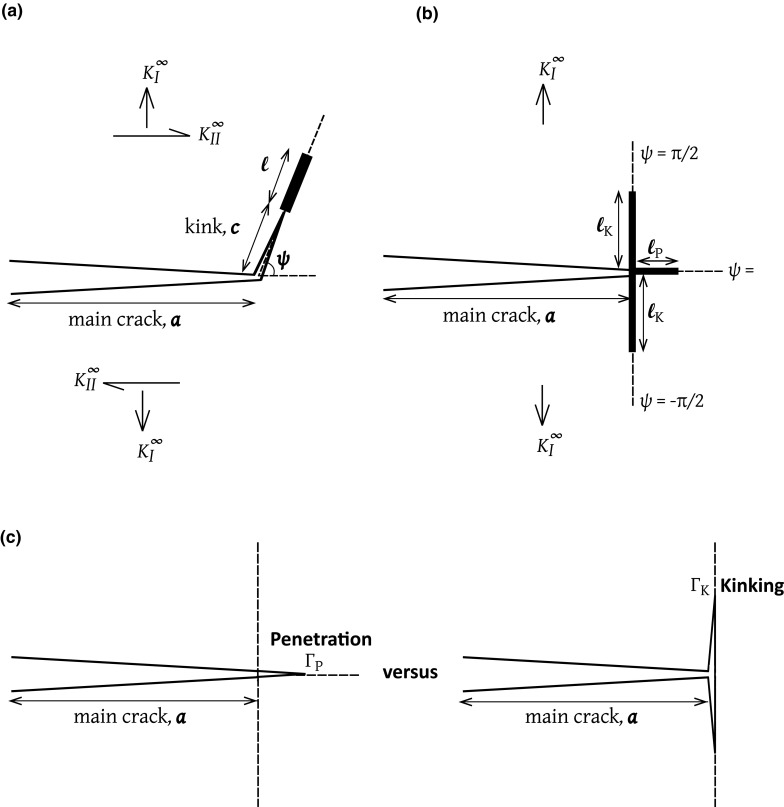
Crack kinking in isotropic solids. **a** General case as considered by He and Hutchinson (1989), **b** $c=0$ and $\psi =0,\pm \pi /2$ as considered by Noselli et al. (2013) and **c** penetration along $\psi =0$ and kinking along $\psi =\pm \pi /2$ of the parent crack at the interface

Consider crack advance in an anisotropic solid such as pine wood. Mode I crack growth *along* the grain occurs at a fracture toughness of $0.3\,\hbox {MPa}\,\sqrt{\mathrm{m}}$; this is much lower than the fracture toughness for crack growth *across* the grain ($3.6\,\hbox {MPa}\,\sqrt{\mathrm{m}}$). Consequently, pine wood has the tendency to split along the grain (Ashby et al. 1985). The correlation between crack deflection and the macroscopic toughness is attributed to the *strong anisotropy of toughness and of elastic properties* of pine wood, and this feature is common to most modern composites such as the fibre/matrix laminates. A literature has developed on kinking in isotropic elastic solids, with an emphasis on the sensitivity of kinking direction to relative *toughness* along different directions (He and Hutchinson 1989; Martinez and Gupta 1994; He et al. 1994b). The case of kinking in a solid with *anisotropic elastic properties* has not been addressed and this is the main purpose of the present study. Consider the prototypical problem of crack kinking from the tip of a parent crack of length *a*, as shown in Fig. 1a. The parent crack is subjected to macroscopic mixed-mode loading, as dictated by a combined remote mode I stress intensity factor $K_{I}^{\infty }$ and a mode II stress intensity $K_{II}^{\infty }$. In order to determine the direction of kinking, we place a daughter crack of length *c* at an orientation $\psi$ to the main cracking plane, and a cohesive zone of length $\ell$ at the tip of the daughter crack. In general, the traction versus crack opening law of the cohesive zone can have both opening and shear components, and we can assume that the traction drops to zero when a critical displacement jump across the cohesive zone is attained. The challenge is to determine the kinking orientation $\psi$ and the critical values of $(K_{I}^{\infty }, K_{II}^{\infty })$ for kink crack growth due to attainment of the critical displacement jump across the cohesive zone.

The problem stated above contains many geometric and material parameters and has been tackled so far for various limiting cases. Hutchinson and co-workers (He and Hutchinson 1989; He et al. 1994a, b; Suo 1990) have considered the case where $\ell /c \rightarrow 0$, such that a finite toughness exists at the tip of the daughter crack. Alternatively, we can consider the case where $c/\ell \rightarrow 0$ such that a cohesive zone exists at the tip of the main crack. This has been analysed by Parmigiani and Thouless (2006) for the case of a crack in a coating on a dissimilar elastic substrate. They supposed that three cohesive zones can exist simultaneously at $\psi =0, \pm \pi /2$ and assumed that the inclined cohesive zones (at $\psi =\pm \pi /2$) carry both shear and normal traction. A similar analysis for $c=0$ was performed more recently by Noselli et al. (2013) for the case of a semi-infinite crack ($a=\infty$) subjected to remote mode I loading and cohesive zones placed at $\psi =0, \pm \pi /2$, see Fig. 1b. The associated kinking criteria, as developed by Hutchinson and co-workers and by the more recent contributions (Parmigiani and Thouless 2006; Noselli et al. 2013), differ due to the fact that different physical cases are under consideration; the former is more relevant to kinking in brittle ceramic composites, whereas the latter is more relevant to ductile materials where strength and toughness both play a role in crack initiation and growth. A brief review of kinking criteria is now presented.

### Kinking criterion based on the energetics of a small kink at the main crack tip

Consider two special cases of the general problem in Fig. 1a: (1) the daughter crack is colinear with the parent crack ($\psi =0$) and (2) the daughter crack is orthogonal to the main cracking plane $\psi =\pm 90^{\circ }$, see Fig. 1c. He and Hutchinson (1989) and Martinez and Gupta (1994) obtained the energy release rates *G* for these two alternative configurations. Write $G_{\mathrm{P}}$ as the energy release rate for crack penetration and $G_{\mathrm{K}}$ as the energy release rate at the tip of each kink crack at $\psi =\pm 90^{\circ }$ to the main cracking plane. And, denote the work of fracture for crack penetration and kinking by $\Gamma _{\mathrm{P}}$ and $\Gamma _{\mathrm{K}}$, respectively. Then, the energy criteria for crack kinking reads (He and Hutchinson 1989)

1 $$\begin{aligned} \dfrac{\Gamma _{\mathrm{P}}}{\Gamma _{\mathrm{K}}} > \dfrac{G_{\mathrm{P}}}{G_{\mathrm{K}}} \end{aligned}$$

Elastic stress analysis predicts that the ratio $G_{\mathrm{P}}/G_{\mathrm{K}}=3.8$ for a crack in a homogeneous and isotropic solid. Accordingly, (1) gives the condition for crack kinking at an interface in an isotropic solid as: ${\Gamma _{\mathrm{P}}}/{\Gamma _{\mathrm{K}}} > 3.8$. Subsequently, Suo et al. (1991) and Wang (1994) considered crack kinking in elastic-brittle, fibre-reinforced composites with a weak fibre/matrix interface. They obtained the values of $G_{\mathrm{P}}/G_{\mathrm{K}}$ for a homogeneous but orthotropic solid in terms of the two elastic parameters $\lambda$ and $\rho$ (which are functions of the orthotropic elastic constants, and are defined in a later section of the current paper) as

2 $$\begin{aligned} \dfrac{G_{\mathrm{P}}}{G_{\mathrm{K}}} = c(\rho ) \lambda ^{1/4} \end{aligned}$$

where $c(\rho )$ is a non-dimensional function of $\rho$. In the limiting case of an isotropic solid ($\lambda = \rho =1$), the He and Hutchinson (1989) solution is recovered.

**Fig. 2:**
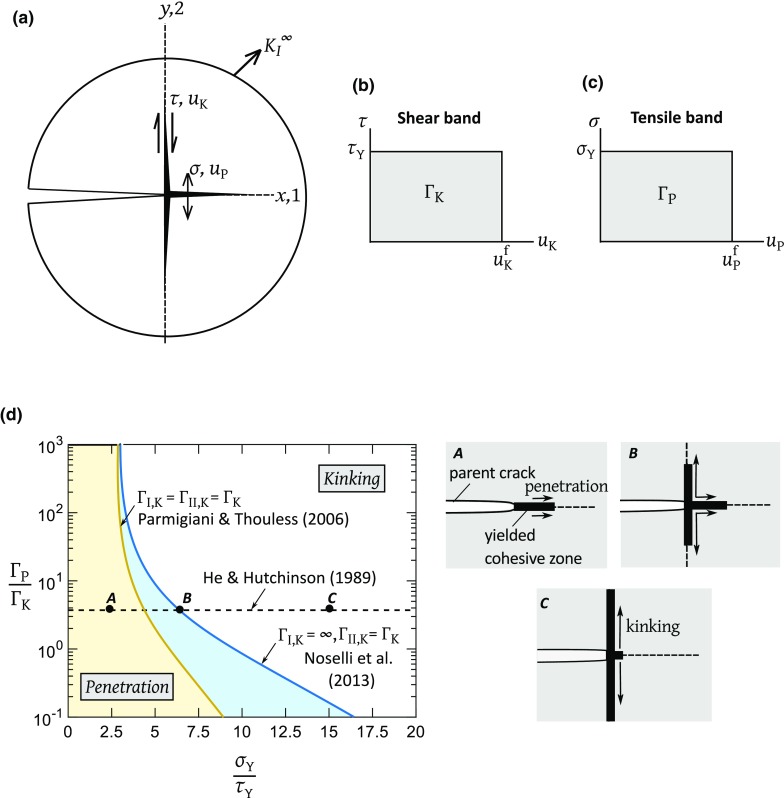
Asymptotic K-field with ductile interfaces at the crack tip. **a** Geometry of the crack and co-ordinate reference frame. Constitutive laws for, **b** shear band and, **c** tensile band. **d** Crack path selection map as obtained from the analyses of Noselli et al. (2013) and Parmigiani and Thouless (2006) for the isotropic case; the inset illustrates the mode of initial crack growth and the relative extent of the active tensile and shear cohesive zones for 3 points *A*, *B*, *C* as predicted by Noselli et al. (2013)

### Kinking criterion based on cohesive zones at the main crack tip

Parmigiani and Thouless (2006) and Noselli et al. (2013) have used finite element simulations to explore crack path selection in an elastic, isotropic solid by assuming the co-existence of cohesive zones directly ahead of the main crack tip ($\psi =0$) and in the kinking direction ($\psi = \pm \pi /2$), as depicted in Fig. 1b. While Parmigiani and Thouless (2006) studied the preferred direction of crack growth of a crack in the surface coating of an elastic solid subjected to remote uniform tensile strain orthogonal to the main crack, Noselli et al. (2013) considered the asymptotic boundary value problem of a semi-infinite parent crack subjected to remote mode I loading as shown in Fig. 2a. A number of different approaches can be adopted for modelling the behaviour of a cohesive zone in the kinking direction under mixed-mode loading. For example, the normal and shear displacements can be combined into a single parameter that is used in a traction-separation law to indicate overall load-bearing capacity. In contrast, Parmigiani and Thouless (2006) used separate independent laws in mode I and mode II. Their mode I toughness $\Gamma _{\mathrm{I,K}}$ is the area under the tensile traction versus opening curve, and their mode II toughness $\Gamma _{\mathrm{II,K}}$ is the area under the shear traction versus shear displacement curve. Since the traction-separation laws are prescribed independently in modes I and II, they are coupled only through a mixed-mode failure criterion. Parmigiani and Thouless (2006) defined the mode I energy release rate $G_{\mathrm{I,K}}$ as the area under the tensile traction versus opening curve up to the current time, and likewise the mode II energy release rate $G_{\mathrm{II,K}}$ as the area under the shear traction versus sliding displacement curve up to the current time. Crack growth occurs when the following linear fracture criterion is satisfied:

3 $$\begin{aligned} \dfrac{G_{\mathrm{I,K}}}{\Gamma _{\mathrm{I,K}}} + \dfrac{G_{\mathrm{II,K}}}{\Gamma _{\mathrm{II,K}}} =1 \end{aligned}$$

Parmigiani and Thouless (2006) further assumed that the magnitudes of strength and toughness in modes I and II are identical such that $\sigma _{\mathrm{KY}}=\tau _{\mathrm{KY}}=\tau _{\mathrm{Y}}$ and $\Gamma _{\mathrm{I,K}}=\Gamma _{\mathrm{II,K}}=\Gamma _{\mathrm{K}}$. In contrast, Noselli et al. (2013) assumed a pure mode II sliding response for the shear bands along $\psi = \pm \pi /2$ such that ($\sigma _{\mathrm{KY}}, \tau _{\mathrm{KY}}) =(\infty , \tau _{\mathrm{Y}}$) and ($\Gamma _{\mathrm{I,K}},\Gamma _{\mathrm{II,K}})=(\infty ,\Gamma _{\mathrm{K}}$). The traction versus separation law for the shear bands along $\psi = \pm \pi /2$ is depicted in Fig. 2b. Additionally, a tensile band of strength $\sigma _{\mathrm{Y}}$ and toughness $\Gamma _{\mathrm{P}}$ exists directly ahead of the parent crack along $\psi =0$; this is shown in Fig. 2c. Both Parmigiani and Thouless (2006) and Noselli et al. (2013) explored crack penetration versus crack kinking as a function of the relative strength and relative toughness of the two interfaces. Figure 2d shows a map of penetration versus kinking with axes of interface strength ratio ${\sigma _{\mathrm{Y}}}/{\tau _{\mathrm{Y}}}$and interface toughness ratio $\Gamma _{\mathrm{P}}/\Gamma _{\mathrm{K}}$ from both studies.

### Comparison of the two kinking criteria

The crack kinking criterion prescribed by He and Hutchinson (1989) is included in Fig. 2d for the purpose of comparison. The following differences are noted between the two approaches discussed in Sects. 1.1 and 1.2:

1. The crack path selection criterion by He and Hutchinson (1989) is purely energetic in nature; it is obtained by calculating the energy release rate at the tip of a small $90^{\circ }$ kink of a main crack. In contrast, the cohesive zone approach of Parmigiani and Thouless (2006) and Noselli et al. (2013) includes both the fracture strength and fracture energy of the two interfaces in determining the active fracture path, and it assumes the co-existence of damage ahead of the main crack and in kinking direction.
2. According to the energy-based criterion of He and Hutchinson (1989), crack kinking occurs when ${\Gamma _{\mathrm{P}}}/{\Gamma _{\mathrm{K}}}$ $> 3.8$, see Fig. 2d. In contrast, the predictions from the cohesive zone models of Parmigiani and Thouless (2006) and Noselli et al. (2013) indicate that the crack path for a given ${\Gamma _{\mathrm{P}}}/{\Gamma _{\mathrm{K}}}$ also depends on the interface strength ratio ${\sigma _{\mathrm{Y}}}/{\tau _{\mathrm{Y}}}$. Consider, for example, ${\Gamma _{\mathrm{P}}}/{\Gamma _{\mathrm{K}}}$ = 3.8. For ${\sigma _{\mathrm{Y}}}/{\tau _{\mathrm{Y}}}$ $< 6.3$, such as ${\sigma _{\mathrm{Y}}}/{\tau _{\mathrm{Y}}}$ $=2.5$ (labelled *A* in Fig. 2d), crack growth occurs via penetration into the tensile band. For ${\sigma _{\mathrm{Y}}}/{\tau _{\mathrm{Y}}}$ $> 6.3$, such as ${\sigma _{\mathrm{Y}}}/{\tau _{\mathrm{Y}}}$= 15 (labelled *C*), the crack-tip will kink. For the choice of (${\Gamma _{\mathrm{P}}}/{\Gamma _{\mathrm{K}}}$ , ${\sigma _{\mathrm{Y}}}/{\tau _{\mathrm{Y}}}$)= (3.8, 6.3), simultaneous crack penetration and kinking occur (labelled *B*) in the prediction of Noselli et al. (2013).
3. The finite mode I toughness of the kink bands along $\psi = \pm \pi /2$ in the Parmigiani and Thouless (2006) study enlarges the zone of kinking compared to the prediction of Noselli et al. (2013).

In the current study, we shall re-visit the regimes of crack path selection for the case when the solid material is elastic and orthotropic.

### Scope of study

The main objective of this study is to predict the macroscopic mode I toughness of an *orthotropic composite* when two forms of damage co-exist: (1) a tensile cohesive zone directly ahead of the main crack-tip, and (2) a shear cohesive zone along a kinking direction orthogonal to the plane of parent crack, see Fig. 2a.

The cohesive zone approach is used to obtain: (1) a criterion for crack path selection between crack penetration and kinking in terms of the relative strength and relative toughness along each direction, (2) the degree of macroscopic mode I toughening associated with the two fracture paths viz., penetration and kinking, and (3) the extent of the damage zones at failure, for selected values of material orthotropy.

## An orthotropic 2D solid

For a general anisotropic material, the elastic constitutive relation, in the Cartesian frame (*x*, *y*, *z*) of Fig. 2a, has the following vector-matrix form

4 $$\begin{aligned} \varepsilon _{i} = \sum \limits _{j=1}^{6} S_{ij} \sigma _{j},\quad i= \text {1 to 6} \end{aligned}$$

where $\{ \varepsilon _{i} \} = \{ \varepsilon _{x}, \varepsilon _{y}, \varepsilon _{z}, \gamma _{yz}, \gamma _{xz}, \gamma _{xy} \}^{T}$, $\{ \sigma _{i} \} = \{ \sigma _{x}, \sigma _{y}, \sigma _{z}, \tau _{yz}, \tau _{xz}, \tau _{xy} \}^{T}$, and $\left[ S_{ij} \right]$ is a $6\times 6$ compliance matrix with 12 independent constants. When the material has an elastic symmetry plane normal to $z-$axis, the stress versus strain relation for deformation in the (*x*, *y*) plane can be reduced to (Lekhnitskii et al. 1968)

5 $$\begin{aligned} \varepsilon _{i} = \sum \limits _{j=1,2,6} A_{ij} \sigma _{j}, \quad i=1, 2, 6 \end{aligned}$$

where

6 $$\begin{aligned} A_{ij} = {\left\{ \begin{array}{ll} S_{ij}, \quad \text {for plane stress} \\ S_{ij}-\dfrac{S_{i3}S_{j3}}{S_{33}}, \quad \text {for plane strain,} \quad i,j=1,2,6 \end{array}\right. } \end{aligned}$$

Further, if the material is orthotropic with *x* and *y* axes coincident with the principal axes of the material, there are only four independent constants $A_{11}$, $A_{12}=A_{21}$, $A_{22}$ and $A_{66}$, but $A_{16}=A_{26}=0$. In this case, the non-zero compliances $S_{ij}$ are related to the engineering constants by

7 $$\begin{aligned} S_{11}= & {} \dfrac{1}{E_{x}},\quad S_{22}=\dfrac{1}{E_{y}},\quad S_{33}=\dfrac{1}{E_{z}}\nonumber \\ S_{12}= & {} -\dfrac{\nu _{xy}}{E_{x}}=S_{21}=-\dfrac{\nu _{yx}}{E_{y}} \nonumber \\ S_{13}= & {} -\dfrac{\nu _{xz}}{E_{x}}=S_{31}=-\dfrac{\nu _{zx}}{E_{z}} \nonumber \\ S_{23}= & {} -\dfrac{\nu _{yz}}{E_{y}}=S_{32}=-\dfrac{\nu _{zy}}{E_{z}} \nonumber \\ S_{66}= & {} \dfrac{1}{G_{xy}} \end{aligned}$$

**Fig. 3:**
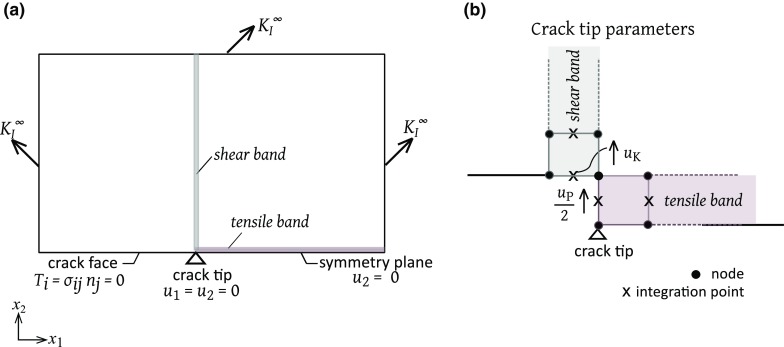
Schematic of the FE implementation: **a** boundary conditions and **b** crack tip parameters

The plane strain mode I displacement field $(u_{1},u_{2})$ in a small annulus surrounding the tip of a semi-infinite crack depends on the polar co-ordinates $(r,\theta )$ according to Sih et al. (1965)

8 $$\begin{aligned} \begin{aligned} u_{1}&= K_{\mathrm{I}}^{\infty } \sqrt{\dfrac{2r}{\pi }}\; \mathfrak {R}\text {e}\\&\left[ \dfrac{1}{\mu _{1}-\mu _{2}} \left( \mu _{1}p_{2} \sqrt{\cos \theta + \mu _{2} \sin \theta } - \mu _{2}p_{1} \sqrt{\cos \theta + \mu _{1} \sin \theta }\right) \right] \\ u_{2}&= K_{\mathrm{I}}^{\infty } \sqrt{\dfrac{2r}{\pi }}\; \mathfrak {R}\text {e} \\&\left[ \dfrac{1}{\mu _{1}-\mu _{2}} \left( \mu _{1}q_{2} \sqrt{\cos \theta + \mu _{2} \sin \theta } - \mu _{2}q_{1} \sqrt{\cos \theta + \mu _{1} \sin \theta }\right) \right] \\ \end{aligned} \end{aligned}$$

where $K_{\mathrm{I}}^{\infty }$ is the mode I stress intensity factor and the constants $(\mu _{i}, p_{i}, q_{i})$ for $i=1,2$ are related to the components $A_{ij}$ of the orthotropic solid as given by Sih et al. (1965).

Suo (1990) has shown that the stress state within an orthotropic solid under plane strain deformation depends only on two non-dimensional elastic parameters $\lambda$ and $\rho$ as given by

9 $$\begin{aligned} \lambda = \dfrac{A_{11}}{A_{22}} \quad \text {and} \quad \rho = \dfrac{2A_{12}+A_{66}}{2 \sqrt{A_{11}A_{22}}} \end{aligned}$$

The parameters $\lambda$ and $\rho$ quantify the anisotropy of the solid. For example, $\lambda =\rho =$1 denotes isotropy and $\lambda =1$, $\rho \ne 1$ denotes transverse isotropy. Positive definiteness of the strain energy density function implies $\lambda>$0 and $\rho >-$1. In order to highlight the effect of orthotropy on crack kinking, we show results for selected values of $\lambda$ and $\rho$ that suitably represent three classes of composites:

1. An orthotropic solid with ($\lambda ,\rho$) = (20, 5) represents unidirectional laminate made from carbon fibres and an epoxy matrix, such as IM7/8552, with lamina properties $E_{1}=E_{x}=E_{z}=$ 8.9 GPa, $E_{2}=E_{y}=162$ GPa, $G_{12}=G_{xy}=4$ GPa, $\nu _{21}=\nu _{yx}=\nu _{yz}=0.3$ and $\nu _{13}=\nu _{xz}=0.5$, as taken from Tan et al. (2016).
2. An orthotropic solid with ($\lambda ,\rho$) = (1, 10) represents a cross-ply laminate, again made from carbon fibres and an epoxy matrix.
3. An isotropic solid is given by ($\lambda ,\rho$) = (1, 1).

## Numerical method

The sensitivity of crack kinking to elastic anisotropy and to the degree of anisotropy in strength and toughness is explored for the orthotropic solid with crack tip cohesive zones as defined in Fig. 2b, c. A boundary layer formulation is adopted such that an outer mode I K-field is applied to a pre-cracked specimen, see Fig. 2a. The parent crack has (1) a *tensile cohesive zone* directly ahead of it ($\psi =0$) and (2) a *shear cohesive zone* emanating symmetrically at $\psi =\pm 90^{\circ }$.

Consider first the shear band at $\psi =\pm 90^{\circ }$. We follow the approach of Noselli et al. (2013) and assume that the shear band has a strength $\tau _{\mathrm{Y}}$  and a failure displacement $u_{\mathrm{K}}^{\mathrm{f}}$, see Fig. 2b. A high value of stiffness is specified in the opening mode of the shear band so that the material on either side of the shear band can only slide. The shear band thus acts as a bridged pure mode II crack with a strength $\tau _{\mathrm{Y}}$ and a toughness $\Gamma _{\mathrm{K}}=\tau _{\mathrm{Y}}u_{\mathrm{K}}^{\mathrm{f}}$. Likewise, we represent the tensile damage zone ahead of the main crack tip by an ideally-plastic cohesive zone of finite yield strength $\sigma _{\mathrm{Y}}$  and failure displacement $u_{\mathrm{P}}^{\mathrm{f}}$, as shown in Fig. 2c. Symmetry dictates that the shear stress in the tensile band vanishes so that it behaves as a bridged pure mode I crack. The characteristic quantities of the tensile band are the cohesive strength $\sigma _{\mathrm{Y}}$ and toughness $\Gamma _{\mathrm{P}}=\sigma _{\mathrm{Y}}u_{\mathrm{P}}^{\mathrm{f}}$.

### The finite element formulation

The macroscopic toughness of the orthotropic composite is calculated from the asymptotic K-field boundary value problem using the commercial finite element (FE) package ABAQUS v6.14. For each choice of material orthotropy as listed in Sect. 2, displacement field (8) associated with a mode I K is applied to the boundary of a rectangular mesh. The mesh comprises 8-noded linear elements and is constrained in the out-of-plane direction to simulate plane strain ($\varepsilon _{33}=0$). Figure 3a illustrates the FE boundary value problem, and Fig. 3b depicts the crack-tip parameters $u_{\mathrm{P}}$ and $u_{\mathrm{K}}$ of the tensile and shear bands, respectively.

**Fig. 4:**
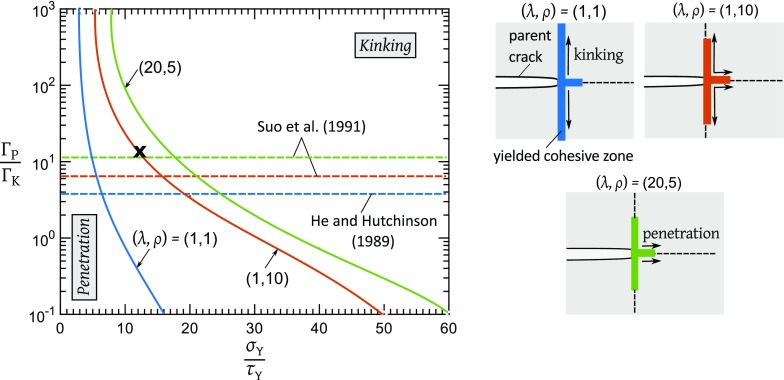
Regimes of dominance of crack penetration versus $90^\mathrm{o}$ crack kinking on a map of relative toughness versus relative strength. The insets illustrate the mode of initial crack growth at the point marked X on the map for the three choices of orthotropy and show the relative extent of the plastic zones in each case

The tensile band and the shear bands are modelled as zero thickness cohesive elements (type COH3D8 in ABAQUS). The elastic, ideally plastic cohesive zone laws are implemented by means of a user subroutine UMAT. Define the length of the active tensile cohesive zone as $\ell _{\mathrm{P}}^{\mathrm{f}}$ at the onset of the crack growth, and the corresponding shear zone length as $\ell _{\mathrm{k}}^{\mathrm{f}}$. An extensive mesh sensitivity study was performed such that the active cohesive zones extended over at least 10 elements, and the finite element mesh extended a distance of at least 10 times the active cohesive zone length to ensure small scale yielding.

### Criterion for crack path selection

The parent crack of Fig. 2a is loaded by a remote mode I stress intensity factor $K_{I}^{\infty }$ or by the equivalent energy release rate $G^{\infty }$, where $G^{\infty }$ is related to $K_{I}^{\infty }$ via (13) as defined later in the paper. In the absence of the shear bands, the macroscopic fracture toughness $G^{\infty }_{\mathrm{f}}$ for crack penetration (or self-similar extension) is given by $G^{\infty }_{\mathrm{f}}=\Gamma _{\mathrm{P}}$. But, when tensile and shear bands co-exist, the macroscopic toughness is dictated by the cohesive zone that fails first.

We shall show below that remote mode I loading always activates the yield of tensile cohesive zone but does not trigger shear yielding along the kink zones if ${\tau _{\mathrm{Y}}}/{\sigma _{\mathrm{Y}}}$ exceeds a critical value (which depends upon the degree of orthotropy). Consider the case where remote mode I loading activates both the tensile and shear cohesive zones. The opening at the mouth of the tensile cohesive zone $u_{\mathrm{P}}$ scales linearly with $G^{\infty }$ and is of the form

10 $$\begin{aligned} {u_{\mathrm{P}}}= \dfrac{G^{\infty }}{\sigma _{\mathrm{Y}}f_{\mathrm{1}}} \end{aligned}$$

where the non-dimensional function $f_{\mathrm{1}}$ depends upon $({\sigma _{\mathrm{Y}}}/{\tau _{\mathrm{Y}}}, \lambda , \rho )$ and is obtained from the FE simulation. Likewise, the maximum value of the shear displacement for the inclined shear bands is

11 $$\begin{aligned} {u_{\mathrm{K}}}= \dfrac{G^{\infty }}{\tau _{\mathrm{Y}}f_{\mathrm{2}}} \end{aligned}$$

where $f_{\mathrm{2}}=f_{\mathrm{2}}({\sigma _{\mathrm{Y}}}/{\tau _{\mathrm{Y}}}, \lambda , \rho )$ is also determined by the FE simulation.

Crack penetration occurs when $u_{\mathrm{P}}=u_{\mathrm{P}}^{\mathrm{f}}$ and $u_{\mathrm{K}}<u_{\mathrm{K}}^{\mathrm{f}}$; alternatively, kinking occurs when $u_{\mathrm{K}}=u_{\mathrm{K}}^{\mathrm{f}}$ and $u_{\mathrm{P}}<u_{\mathrm{P}}^{\mathrm{f}}$. The boundary between penetration and kinking occurs by setting $u_{\mathrm{P}}=u_{\mathrm{P}}^{\mathrm{f}}=\Gamma _{\mathrm{P}}/\sigma _{\mathrm{Y}}$ and $u_{\mathrm{K}}=u_{\mathrm{K}}^{\mathrm{f}}=\Gamma _{\mathrm{K}}/\tau _{\mathrm{Y}}$ to give

12 $$\begin{aligned} \dfrac{\Gamma _{\mathrm{P}}}{\Gamma _{\mathrm{K}}} = \dfrac{f_{2}}{f_{1}} \end{aligned}$$

upon making use of (10) and (11).

**Fig. 5:**
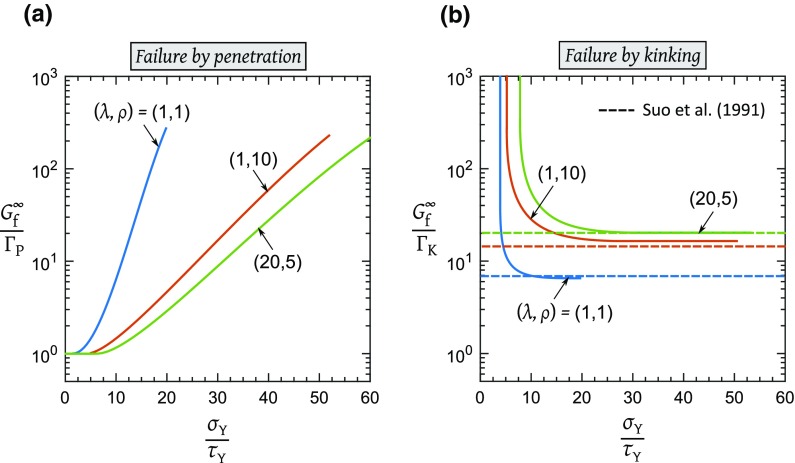
The predicted toughness of each composite as a function of ${\sigma _{\mathrm{Y}}}/{\tau _{\mathrm{Y}}}$  for, **a** failure by penetration, $f_{1}=G^{\infty }_{\mathrm{f}}/\Gamma _{\mathrm{P}}$ and, **b** failure by kinking, $f_{2}=G^{\infty }_{\mathrm{f}}/\Gamma _{\mathrm{K}}$

## Predictions

The following results from the FE simulations are presented in turn: (1) failure mechanism maps showing the regimes of dominance of crack growth by penetration or kinking, (2) the degree of toughening associated with crack penetration versus crack kinking, and (3) the size of the tensile and shear plastic zones at failure.

### Failure mechanism maps

The boundary between crack penetration and kinking is shown in Fig. 4 for the isotropic case $(\lambda , \rho )=(1,1)$ and for the two orthotropic cases $(\lambda , \rho )=(1,10)$ and (20, 5). The following observations are drawn from Fig. 4:

1. There exists a lower bound for the strength ratio $\sigma _{\mathrm{Y}}/\tau _{\mathrm{Y}}$ below which the only mode of failure is crack penetration: $\sigma _{\mathrm{Y}}/\tau _{\mathrm{Y}} \approx 3$ for $(\lambda , \rho )=(1, 1)$, $\sigma _{\mathrm{Y}}/\tau _{\mathrm{Y}} \approx 5$ for $(\lambda , \rho )=(1, 10)$, and $\sigma _{\mathrm{Y}}/\tau _{\mathrm{Y}} \approx 8$ for $(\lambda , \rho )=(20, 5)$.
2. For $\sigma _{\mathrm{Y}}/\tau _{\mathrm{Y}}$ greater than this lower bound value, both crack penetration and kinking are possible depending upon the value of $\Gamma _{\mathrm{P}}/\Gamma _{\mathrm{K}}$ and the degree of orthotropy. Consider for example, cohesive zones of strength ratio $\sigma _{\mathrm{Y}}/\tau _{\mathrm{Y}}=12.5$, and toughness ratio $\Gamma _{\mathrm{P}}/\Gamma _{\mathrm{K}}=12.5$, such that $u_{\mathrm{P}}^{\mathrm{f}}=u_{\mathrm{K}}^{\mathrm{f}}$. This point is labelled X in Fig. 4. The insert of Fig. 4 gives the mode of initial crack growth for this point, and assuming the 3 choices of orthotropy. The extent of the cohesive zones are contrasted in the insert. The main crack kinks by $90^\mathrm{o}$ along the shear band if the bulk solid is isotropic. In contrast, the crack extends straight-ahead in a penetrative mode when the bulk material has high orthotropy such as $(\lambda , \rho )=(20,5)$. Crack growth in a solid of mild orthotropy, with $(\lambda , \rho )=(1,10)$, occurs by simultaneous penetration and kinking. For comparison, the energetic criterion (2) is included in Fig. 4 by making use of the results by Suo et al. (1991). This energetic kinking criterion is independent of the value of $\sigma _{\mathrm{Y}}/\tau _{\mathrm{Y}}$, and in agreement with the analysis of the present study it predicts that the zone of kinking shrinks with increasing $\lambda$ and $\rho$.

**Fig. 6:**
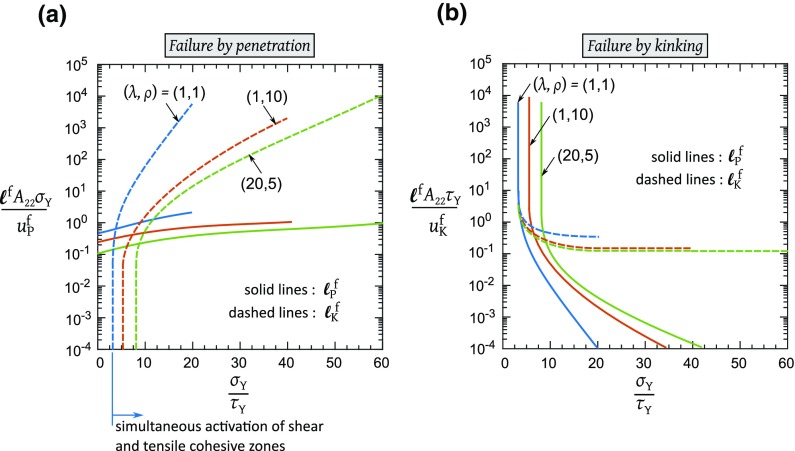
The predicted lengths of the tensile and shear plastic zones at failure as a function of ${\sigma _{\mathrm{Y}}}/{\tau _{\mathrm{Y}}}$  for each composite for, **a** failure by penetration, **b** failure by kinking

### Toughening due to simultaneous activation of cohesive zones

Recall from Sect. 3.2 that the macroscopic toughness of the composite of Fig. 2a depends on whether penetration occurs first or kinking occurs first. When penetration is the active failure mechanism, the macroscopic toughness is given by (10) and when kinking is the active failure mechanism, the macroscopic toughness is given by (11). The non-dimensional functions $f_{1}=G^{\infty }_{\mathrm{f}}/\Gamma _{\mathrm{P}}$ and $f_{2}=G^{\infty }_{\mathrm{f}}/\Gamma _{\mathrm{K}}$, as obtained from the FE simulations, are plotted in Fig. 5a, b respectively, for the three cases of orthotropy. In each of these plots, the applied $K_{I}^{\infty }$ is rewritten in terms of the energy quantity $G^{\infty }$ by making use of the relation (Suo et al. 1991):

13 $$\begin{aligned} G^{\infty } = \sqrt{\dfrac{1+\rho }{2}} \lambda ^{\frac{1}{4}} A_{22} K_{I}^{\infty 2} \end{aligned}$$

For a given set of material properties ($\sigma _{\mathrm{Y}}/\tau _{\mathrm{Y}},u_{\mathrm{P}}^{\mathrm{f}}/u_{\mathrm{K}}^{\mathrm{f}},\lambda ,\rho$), the macroscopic toughness $G^{\infty }_{\mathrm{f}}$ is obtained as follows:

**Step 1.**: Determine from Fig. 4 if the active mode of failure is penetration or kinking.
**Step 2.**: If the critical mechanism of failure is penetration, then the macroscopic toughness $G^{\infty }_{\mathrm{f}}$ is obtained from Fig. 5a in terms of $\Gamma _{\mathrm{P}}$ and ($\sigma _{\mathrm{Y}}/\tau _{\mathrm{Y}},\lambda ,\rho$). If kinking occurs first, then $G^{\infty }_{\mathrm{f}}$ is obtained from Fig. 5b in terms of $\Gamma _{\mathrm{K}}$ and ($\sigma _{\mathrm{Y}}/\tau _{\mathrm{Y}},\lambda ,\rho$).

Note from Fig. 5a that, for all choices of orthotropy, $G^{\infty }_{\mathrm{f}}$ equals $\Gamma _{\mathrm{P}}$ for $\sigma _{\mathrm{Y}}/\tau _{\mathrm{Y}}$ below a threshold, as previously identified in Sect. 4.1. For these low values of $\sigma _{\mathrm{Y}}/\tau _{\mathrm{Y}}$, crack growth is by penetration i.e., by self-similar extension. When $\sigma _{\mathrm{Y}}/\tau _{\mathrm{Y}}$ exceeds the threshold value, the degree of toughening for crack penetration, $G^{\infty }_{\mathrm{f}}/\Gamma _{\mathrm{P}}$ , increases with ${\sigma _{\mathrm{Y}}}/{\tau _{\mathrm{Y}}}$  due to shielding of the crack-tip by both the tensile and shear plastic zones. For example, $G^{\infty }_{\mathrm{f}}/\Gamma _{\mathrm{P}}$ exceeds 100 for $\sigma _{\mathrm{Y}}/\tau _{\mathrm{Y}}>50$, see Fig. 5a. The macroscopic toughness from kinking, $G^{\infty }_{\mathrm{f}}/\Gamma _{\mathrm{K}}$, attains an asymptotic value for large ${\sigma _{\mathrm{Y}}}/{\tau _{\mathrm{Y}}}$. At high ${\sigma _{\mathrm{Y}}}/{\tau _{\mathrm{Y}}}$, the tensile plastic zone becomes vanishingly small, and the asymptotic value is in agreement with that obtained by Suo et al. (1991) for a kinked crack in an elastic orthotropic solid, see Fig. 5b.

The following example demonstrates the combined utility of Figs. 4, 5. Consider again cohesive zones of strength ratio $\sigma _{\mathrm{Y}}/\tau _{\mathrm{Y}}=12.5$, and toughness ratio $\Gamma _{\mathrm{P}}/\Gamma _{\mathrm{K}}=12.5$. The crack-tip kinks if the bulk material is isotropic, and the macroscopic toughness for *kinking* reads from Fig. 5b as $G^{\infty }_{\mathrm{f}}=6.7\Gamma _{\mathrm{K}}$. If instead, the bulk material is orthotropic with $(\lambda , \rho )=(20,5)$, the parent crack extends in a self-similar manner and the macroscopic toughness for *penetration* reads from Fig. 5a as $G^{\infty }_{\mathrm{f}}=1.4\Gamma _{\mathrm{P}} = 17.5\Gamma _{\mathrm{K}}$. For this example, orthotropy results in an enhancement of the macroscopic toughness by a factor of 2.6.

### Extent of yielded cohesive zones at failure

The enhancement of the macroscopic toughness, as given by (10) and (11), is a direct consequence of the extent of simultaneous yielding within the cohesive zones in the tensile and shear bands. It is instructive to plot the plastic zones at failure for each mode of failure. As previously stated, the tensile plastic zone $\ell _{\mathrm{P}}$ is the distance from the crack-tip over which the tensile traction attains the value $\sigma _{\mathrm{Y}}$  whereas the shear plastic zone $\ell _{\mathrm{K}}$ is given by the total height over which the shear traction attains the value $\tau _{\mathrm{Y}}$. Dimensional arguments and linearity of the problem dictate that the extent of plastic zones at failure, $\ell _{\mathrm{P}}^{\mathrm{f}}$  and $\ell _{\mathrm{K}}^{\mathrm{f}}$  are given in terms of the four functions $g_{1}$ through $g_{4}$ , such that

14 $$\begin{aligned} \begin{aligned}&\dfrac{\ell _{\mathrm{P}}^{\mathrm{f}} A_{22} \sigma _{\mathrm{Y}}}{u_{\mathrm{P}}^{\mathrm{f}}} = g_{\mathrm{1}} \left( \dfrac{\sigma _{\mathrm{Y}}}{\tau _{\mathrm{Y}}}, \lambda , \rho \right) \quad \text {and} \\&\dfrac{\ell _{\mathrm{K}}^{\mathrm{f}} A_{22} \sigma _{\mathrm{Y}}}{ u_{\mathrm{P}}^{\mathrm{f}}} = g_{\mathrm{2}} \left( \dfrac{\sigma _{\mathrm{Y}}}{\tau _{\mathrm{Y}}}, \lambda , \rho \right) \end{aligned} \end{aligned}$$

when penetration is the critical failure mechanism, and

15 $$\begin{aligned} \begin{aligned}&\dfrac{\ell _{\mathrm{P}}^{\mathrm{f}} A_{22} \tau _{\mathrm{Y}}}{u_{\mathrm{K}}^{\mathrm{f}}} = g_{\mathrm{3}} \left( \dfrac{\sigma _{\mathrm{Y}}}{\tau _{\mathrm{Y}}}, \lambda , \rho \right) \quad \text {and} \\&\dfrac{\ell _{\mathrm{K}}^{\mathrm{f}} A_{22} \tau _{\mathrm{Y}}}{ u_{\mathrm{K}}^{\mathrm{f}}} = g_{\mathrm{4}} \left( \dfrac{\sigma _{\mathrm{Y}}}{\tau _{\mathrm{Y}}}, \lambda , \rho \right) \end{aligned} \end{aligned}$$

when kinking occurs first. The non-dimensional functions $g_{1}$ through $g_{4}$ follow directly from the FE simulations. In Fig. 6, we plot $\ell _{\mathrm{P}}^{\mathrm{f}}$ and $\ell _{\mathrm{K}}^{\mathrm{f}}$ in terms of the normalized values $g_{1}$ through $g_{4}$, as defined in (14) and (15). Figure 6a gives the plastic zone lengths $(\ell _{\mathrm{P}}^{\mathrm{f}},\ell _{\mathrm{K}}^{\mathrm{f}})$ at failure when penetration is the active mode of failure, and Fig. 6b gives $(\ell _{\mathrm{P}}^{\mathrm{f}},\ell _{\mathrm{K}}^{\mathrm{f}})$ when kinking is the active mode of failure. The predictions for $\ell _{\mathrm{K}}^{\mathrm{f}}$ are terminated at the value of ${\sigma _{\mathrm{Y}}}/{\tau _{\mathrm{Y}}}$ below which no shear yielding occurs. We note that this is the minimum value of ${\sigma _{\mathrm{Y}}}/{\tau _{\mathrm{Y}}}$ (as marked in Fig. 6a for the $\lambda =\rho =1$ case) below which the tensile and shear cohesive zones are not simultaneously activated.

## Concluding discussion

**Fig. 7:**
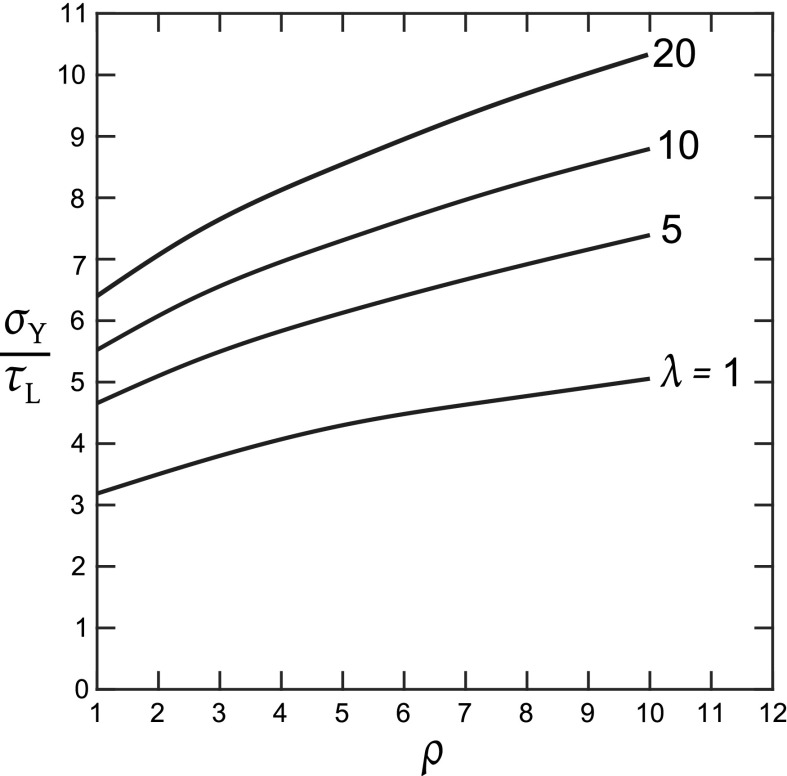
Estimate of the minimum value of ${\sigma _{\mathrm{Y}}}/{\tau _{\mathrm{Y}}}$  for simultaneous yielding in tensile and shear bands

It is seen from Fig. 6 that there exists a minimum value of $\sigma _{\mathrm{Y}}/\tau _{\mathrm{Y}}$ for the simultaneous yielding of tensile and shear cohesive zones, and this value depends on the orthotropic properties of the solid ($\lambda , \rho$). The value of $\sigma _{\mathrm{Y}}/\tau _{\mathrm{Y}}$ that gives rise to the activation of the shear bands is obtained by considering the classical Dugdale problem of a tensile cohesive zone of strength, $\sigma _{\mathrm{Y}}$, in a remote $K_{\mathrm{I}}$ field. However, there is no straightforward analytical solution for the anisotropic Dugdale problem and hence we have used finite element simulations to obtain the level of shear stress $\tau _{\mathrm{L}}$ at the location of a putative shear band. The resulting value of ${\sigma _{\mathrm{Y}}}/{\tau _{\mathrm{L}}}$ is plotted in Fig. 7 as a function of $\rho$ for selected values $\lambda$ in the range of 1–20. For the 3 cases of composite considered in this study, we find from Fig. 7 that $\sigma _{\mathrm{Y}}/\tau _{\mathrm{L}} = \pi$ for $(\lambda , \rho )=(1, 1)$, $\sigma _{\mathrm{Y}}/\tau _{\mathrm{L}} = 5.1$ for $(\lambda , \rho )=(1, 10)$, and $\sigma _{\mathrm{Y}}/\tau _{\mathrm{L}} = 8.5$ for $(\lambda , \rho )=(20, 5)$. The value for the isotropic case is in agreement with the Westergaard solution for the Dugdale problem as given by Tada et al. (1985) and the results of Fig. 7 are consistent with the anisotropic predictions of Fig. 6.

The present study highlights the sensitivity of crack path to the level of elastic orthotropy and to the degree of anisotropy in strength and toughness of a composite. It is generally observed that unidirectional composites such as carbon fibre/epoxy or woods (such as pine) split along the fibre direction. It is clear from Fig. 4 that this tendency to kink/split along the stiff fibre direction is not a consequence of the high stiffness along the fibre direction. Rather, it is a consequence of a large value of $\Gamma _{\mathrm{P}}/\Gamma _{\mathrm{K}}$ and/or large values of $\sigma _{\mathrm{Y}}/\tau _{\mathrm{Y}}$. Our study thus highlights the competition between the effect of elastic anisotropy and toughness/strength anisotropy.
